# Towards pathway-centric cancer therapies via pharmacogenomic profiling analysis of ERK signalling pathway

**DOI:** 10.1186/s40169-015-0066-1

**Published:** 2015-07-28

**Authors:** Haiyun Wang, Xiaoqi Zheng, Teng Fei, Jinzeng Wang, Xujuan Li, Yin Liu, Fan Zhang

**Affiliations:** School of Life Science and Technology, Tongji University, Shanghai, 200092 China; Department of Mathematics, Shanghai Normal University, Shanghai, 200234 China; Department of Biostatistics and Computational Biology, Dana-Faber Cancer Institute and Harvard School of Public Health, Boston, MA 02215 USA; Division of Molecular and Cellular Oncology, Department of Medical Oncology, Dana-Farber Cancer Institute and Harvard Medical School, Boston, MA 02215 USA; Clinical Translational Research Center, Shanghai Pulmonary Hospital, School of Life Science and Technology, Tongji University, Shanghai, 200092 China

**Keywords:** Personalized medicine, ERK signalling pathway, Pathway, Cancer therapies, Pharmacogenomic

## Abstract

**Background:**

Genomic heterogeneity in human cancers complicates gene-centric personalized medicine. Malignant tumors often share a core group of pathways that are perturbed by diverse genetic mutations. Therefore, one possible solution to overcome the heterogeneity challenge is a shift from gene-centric to pathway-centric therapies. Pathway-centric perspectives, which underscore the need to understand key pathways and their critical properties, could address the complexity of cancer heterogeneity better than gene-centric approaches to aid cancer drug discovery and therapy.

**Methods:**

We used large-scale pharmacogenomic profiling data provided by the Cancer Genome Project of the Wellcome Trust Sanger Institute and the Cancer Cell Line Encyclopedia. In a systematic in silico investigation of ERK signalling pathway components and topological structures determines their influences on pathway activity and targeted therapies. Mann–Whitney U test was used to identify gene alterations associated with drug sensitivity with *p* values and Benjamini–Hochberg correction for multiple hypotheses testing.

**Results:**

The analysis demonstrated that genetic alterations were crucial to activation of effector pathway and subsequent tumorigenesis, however drug sensitivity suffered from both drug effector and non-effector pathways, which were determined by not only underlying genomic alterations, but also interplay and topological relationship of components in pathway, suggesting that the combinatorial targets of key nodes in perturbed pathways may yield better treatment outcome. Furthermore, we proposed a model to provide a more comprehensive insight and understanding of pathway-centric cancer therapies.

**Conclusions:**

Our study provides a holistic view of factors influencing drug sensitivity and sheds light on pathway-centric cancer therapies.

**Electronic supplementary material:**

The online version of this article (doi:10.1186/s40169-015-0066-1) contains supplementary material, which is available to authorized users.

## Background

The principle of personalized cancer therapy is to develop therapeutic strategies targeting specific genomic alterations and/or perturbed pathways associated with specific cancers. Much of the ongoing effort has been directed at identifying individual biomarkers of effective therapies and developing anticancer drugs targeting specific genomic alterations [1–4]. However, genomic heterogeneity in human cancers complicates gene-centric personalized medicine. Mutated genes vary widely across individual tumors. Indeed, the cancer genomic landscape proposed by Wood et al. [5] describes tumor heterogeneity as mountains and hills representing frequently and infrequently mutated genes, respectively. Most of these altered genes are mutated only in a small percentage of tumors, and this heterogeneity has been a major obstacle to identify effective cancer therapies for many patients. Moreover, some altered proteins, such as RAS, are difficult to target therapeutically [6]. One possible solution to overcome the heterogeneity challenge is a shift from gene-centric to pathway-centric therapies.

Malignant tumors often share a core group of about a dozen perturbed pathways [7] that regulate specific aspects of oncogenesis and tumor metastasis and thereby affect cell fate determination, cell survival, and genome stability [8]. Pathway-oriented perspectives of cancer have important implications for discovering specialized therapies that broadly target key nodal points of altered pathways rather than targeting specific genetic alterations associated with certain cancers [7–9].

However, before potential pathway-driven therapies can be utilized, it is necessary to understand the key pathways and their critical properties related to cancer. Two vital properties of pathways are components and structures. Pathway components, including genes and proteins, that are altered widely across individual tumors, but they often have overlapping functional consequences. For instance, 44% of squamous cell lung cancers harbor altered genes for squamous cell differentiation, including overexpression and amplification of *SOX2* and *TP63*; loss-of-function of *NOTCH1*, *NOTCH2,* and *ASCL4*; and focal deletions in *FOXP1* [10]. Convergence of these different altered genes into specific cellular functions or pathways implies a possibility to target pathways, rather than individual mutated genes, for effective cancer therapies. Therefore, mapping altered genes into pathways promises to simplify tumor mutation heterogeneity for cancer therapies. Pathway structures, with redundancy and feedback to maintain stable organismal functions, may also impact the therapeutic effect of target drugs. For instance, melanoma and colon cancer patients harboring the same *BRAF* (V600E) mutation respond differently to BRAF inhibitors because colon cancer cells but not melanoma cells gain feedback activation of EGFR and its signaling pathway [11]. Therefore, understanding pathway structures and key nodal points targeted by therapeutic agents is fundamental for pathway-centric cancer therapies.

To develop effective pathway-centric therapies, it is necessary to reveal the impact of perturbed pathways and their topological structures on anticancer therapies. Two independent, large-scale pharmacogenomic datasets are available from the Cancer Genome Project (CGP) [12] and the Cancer Cell Line Encyclopedia (CCLE) [13], and currently they are under utilized to investigate the association between pathway properties and drug sensitivity. In this study, we analyzed high-throughput genomic information and pharmacological profiling of anticancer drugs across hundreds of cell lines using datasets provided by the CGP and CCLE. In particular, we focused on ERK signalling to systematically investigate the influence of gene alterations, and pathway topological structure on pathway activity and drug sensitivity. This represents a proof-of-principle study for modelling of pathway-centric cancer therapies.

## Methods

### Datasets

Gene expression, mutation, and drug sensitivity data were available from two independent large-scale pharmacogenomic studies, CGP (http://www.cancerrxgene.org) and CCLE (http://www.broadinstitute.org/ccle/). CGP included nearly 800 human tumour cell lines with full exon sequencing of 64 commonly mutated cancer genes, genome-wide analysis of copy number variation, analysis of seven commonly rearranged cancer genes, expression profiling of 14,500 genes, and pharmacological profiling for nearly 500 of 130 compounds. Human tumour cell lines cover the spectrum of common and rare types of adults and childhood cancers of epithelial, mesenchymal and haematopoietic origin. A range of 275–507 cell lines was screened per drug (mean = 368 cell lines per drug). Cells were treated with nine concentrations of drug for 72 h before measuring cell number relative to controls [12]. The natural logarithm of the half maximal inhibitory concentration (IC50) represented drug sensitivity. CCLE included around ~500 human cancer cell lines profiled for mutations of >1,600 genes, copy number variation of ~20,000 genes, expression profiling of ~20,000 genes, and pharmacological profiling of 24 compounds. These cell lines also cover multiple cancer types. Eight-point dose–response curves were generated for 24 anticancer drugs using an automated compound-screening platform [13]. Drug response was represented by the area under the dose response curve, or activity area [14].

### Data analysis

We classified cell lines into two groups based on the presence or absence of genomic alterations. Mann–Whitney U test was used to identify gene alterations associated with drug sensitivity with *p* values and Benjamini–Hochberg correction for multiple hypotheses testing. Then, as a case study, we focused on drugs targeting the ERK signalling pathway (EGFR inhibitors, BRAF inhibitors, and MEK inhibitors) and oncogenes associated with ERK signalling, including *EGFR*, *BRAF*, *KRAS*, and *NRAS*. By integrating pathway structures, we unravelled the association between pathway structures and drug sensitivity. Our findings were further validated using an independent pharmacological study CCLE.

## Results

For this study, we conducted the statistical analysis on the CGP dataset and validation on the CCLE dataset. CGP dataset consists of 64 commonly mutated cancer genes, as well as their copy number variation, seven commonly rearranged cancer genes and drug sensitivity to 130 compounds in nearly 800 cell lines. The results obtained from CGP were successively validated in the independent CCLE dataset, including around 500 human cancer cell lines profiled for mutations of >1,600 genes and pharmacological profiling of 24 compounds.

### Genomic alterations affect pathway function and drug sensitivity

In CGP data, drug response was presented by the natural logarithm of the half maximal inhibitory concentration (IC50). To identify genes that influence drug sensitivity, we classified cell lines into two groups based on the presence or absence of genomic alterations. Mann–Whitney U test was used to identify gene alterations associated with drug sensitivity with *p* values and Benjamini–Hochberg correction [15] for multiple hypotheses testing (Additional file 1: Table S1, adjusted *p* < 0.1).

**Fig. 1:**
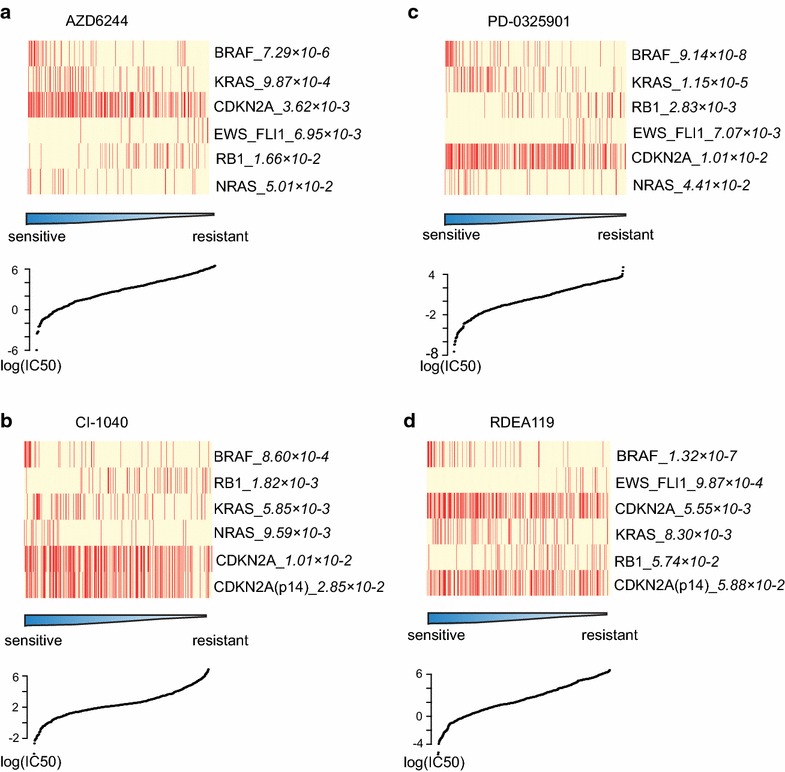
Association between genomic alterations and drug sensitivity. Cells with the indicated mutations were analyzed for sensitivities to the structures of drugs AZD6244 (**a**), CI-1040 (**b**), PD-0325901 (**c**), or RDEA119 (**d**), measured as log (IC50). Each *row* in the heatmap represents a gene and each *column* represents a cell line, with mutations in *red* and wildtype in *yellow*. 445 (**a**), 432 (**b**), 469 (**c**) and 469 (**d**) cell lines covering multiple cancer types were respectively investigated for each drug. Cell lines were ranked by drug sensitivity with a range of −5.83 to 6.39 (**a**), −3.52 to 7.10 (**b**), −7.58 to 4.70 (**c**), and −5.31 to 6.52 (**d**).

One group of drugs that have shown promise in cancer therapy are the MEK inhibitors [16], including AZD6244, CI-1040, PD-0325901, and RDEA119, which inhibit ERK signalling by targeting MEK1/2 kinases. Based on the adjusted *p* value calculated by Mann–Whitney U test, we selected the top six genes mostly associated with drug sensitivity (adjusted *p* < 0.1). Among them, cells with variants of *BRAF*, *KRAS*, *NRAS* and *CDKN2A* were sensitive to MEK inhibitors, while cells with *EWS*-*FLI1* gene translocations or *RB1* mutations were resistant to them (Fig. 1).

*BRAF*, *KRAS* and *NRAS* are key oncogenes in the ERK signalling pathway, and activating mutations in *BRAF*, *KRAS*, and *NRAS* functionally overlap, inducing continuous stimulation of the ERK pathway [17–19]. Since MEK inhibitors inhibit ERK signaling by targeting MEK1/2 kinases, they may effectively treat tumors with *KRAS*, *BRAF*, or *NRAS* mutations [20]. This suggests that drugs are effective if they could successfully repress the ectopic activity of their targeting pathways. Meanwhile, cells harboring genomic aberrations in different pathways—for example, via *EWS*-*FLI1* gene translocations or *RB1* mutations—were resistant to MEK inhibitors. That means drug non-effector pathways could also impact therapeutic effects since they regulate specific aspects of oncogenesis and tumor metastasis as well.

### Topological structures of drug effector pathways influence drug sensitivity

**Fig. 2:**
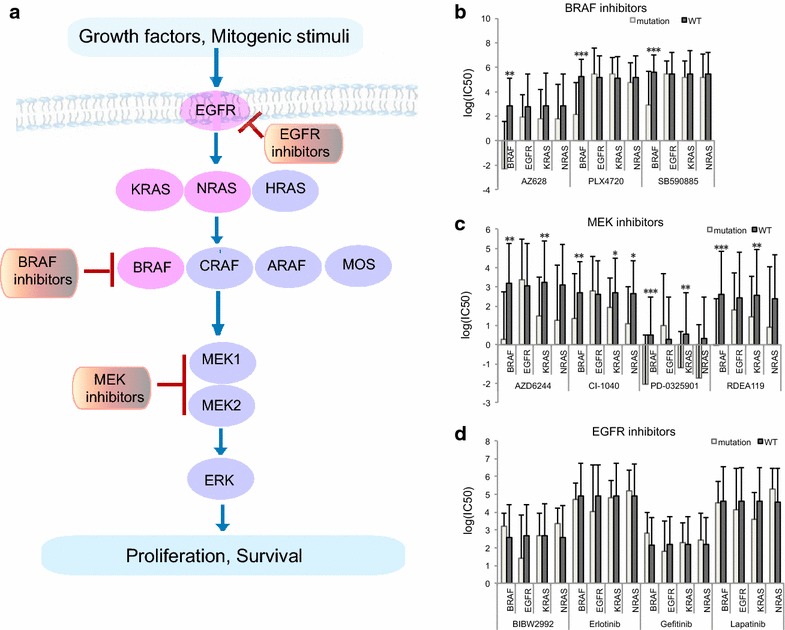
Cells with mutant *EGFR*, *BRAF*, *KRAS*, or *NRAS* respond differently to EGFR inhibitors, BRAF inhibitors, or MEK inhibitors. **a** EGFR inhibitors, BRAF inhibitors, and MEK inhibitors target different components of the ERK signalling pathway, which translates growth factors through signalling components into cell proliferation and survival. *Pink* genes have mutations in the CGP dataset. **b**–**d** Sensitivities of wildtype cells and cells with mutant *EGFR*, *BRAF*, *KRAS*, or *NRAS* to BRAF inhibitors (**b**), MEK inhibitors (**c**), or EGFR inhibitors (**d**), measured as log (IC50). Statistical significance is compared with wildtype. *Adjusted *p* < 0.05; **adjusted *p* < 10^−3^; ***p < 10^−6^.

To investigate how topological pathway structures influence drug effects, we employed the ERK signalling pathway with integration of mutation and drug targets for analysis (Fig. 2a). Since drugs targeting ERK signalling pathway, such as BRAF inhibitors and ERK inhibitors, are single-target compounds rather than multi-target ones, which benefits the association analysis between drug sensitivity and genomic alterations. Moreover, CGP dataset includes a variety of drugs targeting ERK pathway. Drugs associated with this pathway were classified into three groups: EGFR inhibitors, BRAF inhibitors, and MEK inhibitors. The target genes of these inhibitors function upstream, midstream, and downstream, respectively, of the ERK pathway. Then, we focused on known oncogenes in the ERK pathway and used the Mann–Whitney U test to identify associations between pathway structure and drug sensitivity.

Mutations in *EGFR, BRAF, KRAS,* and *NRAS* can lead to acquired and increased ERK signalling activity (Fig. 2a). EGFR inhibitors, MEK inhibitors, and BRAF inhibitors can control the growth of cancer cells by abating extra pathway activity, so effects of these drugs partially depend on their ability to control aberrant activity of the pathway due to activating oncogene mutations. In our analysis, cells with *BRAF* mutations were more sensitive to BRAF inhibitors than cells with *KRAS, NRAS,* or *EGFR* mutations (Fig. 2b). KRAS, NRAS, and EGFR function upstream of BRAF in the signalling pathway. Signals could thus bypass BRAF through ARAF, CRAF, and MOS, which may exert similar functions as BRAF. Therefore, specific targeting of BRAF alone in cells with *KRAS, NRAS,* or *EGFR* mutations fails to inhibit ectopic activation of signals from upstream oncogenic mutations (Fig. 2a).

*KRAS, NRAS,* and *BRAF* mutations are associated with sensitivity to MEK inhibitors, but *EGFR* mutations are not (Fig. 2c). MEK inhibitors target MEK1/2, which are pivotal to activating the downstream pathway. Although BRAF, KRAS, and NRAS are kinases upstream of MEK1/2, they pass signals down through MEK1/2 and do not function through other major signalling branches. Therefore, MEK inhibitors appear to effectively block aberrantly-activated signals from KRAS, NRAS, and BRAF.

Cells harboring *EGFR, KRAS, NRAS,* or *BRAF* mutations displayed limited therapeutic responses to EGFR inhibitors compared with wildtype cells (Fig. 2d). EGFR is the cell-surface receptor upstream of ERK and PI3K signalling pathways, and BRAF, KRAS, and NRAS all function downstream of EGFR. Activating mutations in BRAF, KRAS and NRAS can confer constitutive activation of ERK signalling without stimulation of their upstream signalling, thus EGFR inhibitors are not beneficial for cells with *BRAF, KRAS,* and *NRAS* mutations. A bit surprisingly, cells with *EGFR* mutation were not statistically more sensitive to EGFR inhibitors than cells without *EGFR* mutation for any inhibitors. Moreover, cells with *EGFR* mutation were not more sensitive to BRAF and MEK1/2 inhibitors (Fig. 2b, c). Those results could be due to insufficient *EGFR* mutants in the CGP dataset [10/639 (1.56%)] or redundant pathways other than BRAF or MEK1/2 to transfer ectopic EGFR signals (e.g., PI3K). Our analysis suggests that there might exist the effective targeting nodes as potential pathway-centric therapies, which should locate in the downstream of a pathway and be a unique path for transferring signalling.

### Validation with CCLE dataset

**Fig. 3:**
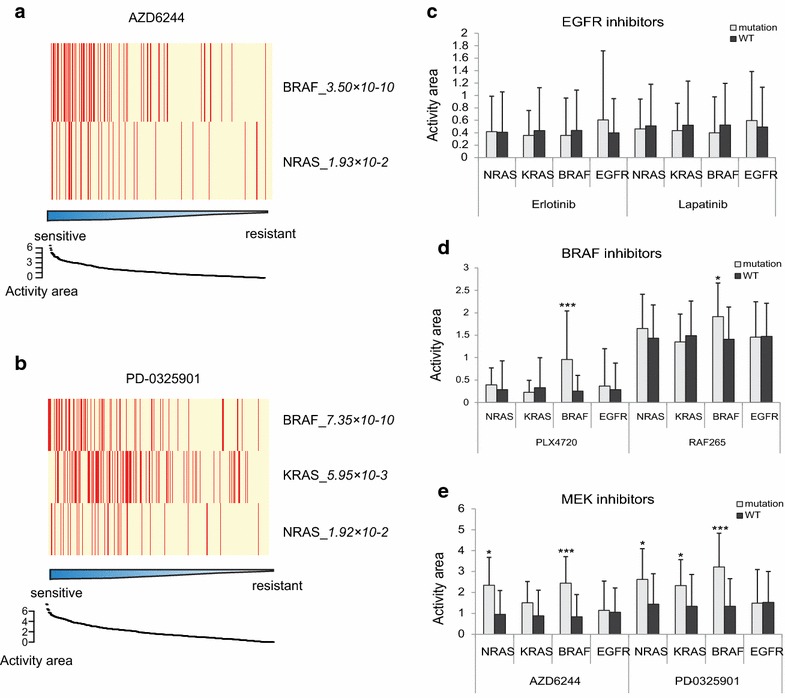
Validation with CCLE dataset. **a**, **b** Responses of cells with *BRAF*, *KRAS*, or *NRAS* mutations to treatment with AZD6244 (**a**) or PD-0325901 (**b**). Each *row* in the heatmap represents a gene and each *column* represents a cell line, with mutations in *red* and wildtype in *yellow*. 450 (**a**) and 451 (**b**) cell lines covering multiple cancer types were respectively investigated for each drug. Cell lines were ranked by drug sensitivity with a range of 0 to 6.49 (**a**), and 0 to 7.38 (**b**). **c**–**e** Responses of cells with mutant *EGFR*, *BRAF*, *KRAS*, or *NRAS* to different anticancer drugs targeting the ERK signalling pathway, measured as activity area. Statistical significance is compared to wildtype. *Adjusted *p* < 0.05; **adjusted *p* < 10^−3^; ***adjusted *p* < 10^−6^.

We used the independent CCLE dataset to confirm our findings. In CCLE data, the IC50 could not be estimated in many cases, as drug concentration necessary to inhibit 50% of growth was not reached. Therefore, different from IC50 in CGP data, drug response in CCLE data was presented by the value of activity area and greater value means higher sensitivity (see “Methods”). In this dataset, *BRAF, KRAS,* and *NRAS* mutations were still sensitive to MEK1/2 inhibitors (Fig. 3a, b).

Further, CCLE analysis from the view of topological structures also showed consistency with our previous CGP findings (Fig. 3c–e). Our analysis further confirmed that MEK inhibitors effectively inhibit ERK signalling by targeting MEK1/2 kinases and are applicable in the treatment of tumors harboring *KRAS, BRAF,* and *NRAS* mutations [20].

### Model of pathway-driven personalized medicine

**Fig. 4:**
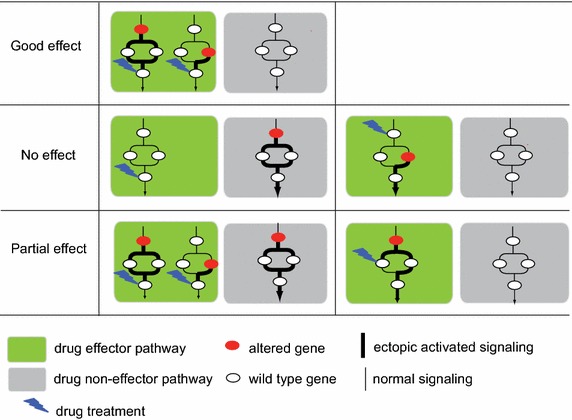
Model of pathway-centric personalized medicine. *Green* and *grey rectangles* indicate drug effector and drug non-effector pathways, respectively; *red* and *white circles* represent individually altered and wildtype genes, respectively; *normal* and *bold lines* indicate normal and ectopic activated pathway signalling, respectively; and *flash signs* indicate drugs targeting specific molecules. One case with good drug effect, two cases with no drug effect, and two cases with partial drug effect are demonstrated.

Based on our analyses, we generated a conceptual model to delineate pathway-driven personalized medicine. In this model (Fig. 4), all pathways might be grouped into drug effector and non-effector pathways. We demonstrated three types of representative cases (cases with good drug effect, cases without drug effect, and cases with partial drug effect) that delineate the association between activity change of perturbed pathways, including drug effector and non-effector pathways, with drug sensitivity.

In the case with good drug effect, drug effector pathways are abnormally activated, which could be caused by two different mutant oncogenes in this pathway. Drug non-effector pathways have normal signalling. In this case, drugs targeting effective targeting nodes of this perturbed pathway usually work well. The effective targeting nodes should be downstream and unique molecules in this pathway. By inhibiting these molecules, drugs are more likely to inhibit the activity of the whole pathway. For instance, MEK inhibitors completely inhibit ERK signalling by targeting MEK1/2 kinases, and are effective in treatment of tumors harboring *KRAS, BRAF,* or *NRAS* mutations. In two cases with no drug effect, the drug is not targeting the perturbed pathway or altered downstream genes gained ectopic activation independent of upstream stimulation, so treatment is ineffective. In two cases with partial drug effect, the first case simulates a tumor with multiple perturbed pathways. Single drugs targeting one pathway are often therapeutically ineffective because other perturbed pathways still provide cellular growth or survival stimulation. In the second case with partial drug effect, the drug could not completely inhibit the activated pathway because its targets were not unique molecules for activation of the downstream pathway. In this case, the drug has little effect on cell growth.

## Discussion

In this study, we systematically investigate ERK signalling pathway components and topological structures that determine their influences on pathway activity and targeted therapies. A tumor is a dynamic entity that evolves by acquiring a series of mutations with advantageous phenotypes, therefore a tumor in general has multiple perturbed pathways. Our analyses demonstrate that genomic alterations may affect multiple pathways, which could be classified as drug effector and non-effector pathways by the way they target the two cancer hallmarks ‘sustaining proliferative signaling’ and ‘evading growth suppressors’ [21]. Both the activity of drug effector pathways and drug non-effector pathways could affect therapeutic effects of target drugs. The researchers [22] have concluded that the idea that the presence of a specific mutation translates into sensitivity or resistance to a particular drug is likely too simplistic, since it does not capture the complexity of the signalling pathways in an individual cancer. We finally generated a conceptual model to delineate pathway-driven personalized medicine. In the third case of this model, pathway-centric personalized cancer therapies can represent combined therapies targeting different perturbed pathways.

Many pathway-centric studies were previously reported [23–27]. For example, Gene expression signature was identified to reflect the activation status of several oncogenic pathways that were deregulated in tumours and clinically relevant associations with disease outcomes. They linked pathway deregulation with sensitivity to therapeutics to guide the use of targeted therapeutics [23]. Genes involved in oxidative stress response and squamous differentiation pathways were found to be frequently altered by mutation or copy number alteration or up-/down- regulation in squamous cell lung cancer [27]. A limitation of these studies is that they relied on limited data or information between drugs and molecules. Our study utilizes two large-scale pharmacogenomic profiles and provides a different view for understanding pathway-centric cancer therapies.

Our analysis showed that cells with EGFR mutation were not statistically more sensitive to EGFR inhibitors than cells without EGFR mutation for any inhibitors. In clinic EGFR inhibitors gefitinib and erlotinib are widely used in non-small-cell lung cancer patients with EGFR mutations for their dramatic efficacy [28–30]. However, clinical studies have shown that a small population of patients with amplified, wild-type EGFR lung cancers also benefit from gefitinib or erlotinib [31, 32]. Our results could be due to insufficient EGFR mutants in the CGP dataset or atypical response of cell lines without EGFR mutations. Moreover, some confounding factors, such as the cancer-type may complicate the drug sensitivity. In addition, the association between NRAS mutation and drug sensitivity are not totally statistically significant in all MEK inhibitors across the two datasets, which also could be due to insufficient NRAS mutants in the datasets. With the availability of more pharmocogenomic data, this statistical analysis will get more power.

Despite the encouraging results, our approach suffers from the following limitations. First, some confounding factors, such as the cancer-type from which a cell line has been originated, or its genomic instability, may contribute to drug sensitivity. Stratification analysis or multivariate analysis could be preferred to determine the association between gene alterations and drug sensitivity. However, when focusing on the individual classes of mutations in the context of ERK signalling pathway, stratification analysis or multivariate analysis could strongly reduce the population size for some test. Therefore, in this study univariate statistical test was used to determine gene alterations contributing to the increased level of resistance/sensitivity to a given drug. This analysis could over-estimate the significance of their association. Second, a recent study revealed significant discordance between CGP and CCLE datasets. These two studies used different experimental protocols. Differences include the pharmacological assay used, the range of drug concentrations tested, and choice of an estimator for summarizing the drug sensitivity [33]. With the availability of more and better quality pharmocogenomic data, our approach will have more reliable findings.

## Conclusions

We applied statistical methods to analyze two recently published pharmacogenomic datasets to investigate pathway components and topological structures and their influences on targeted cancer therapies. The results show that activity of drug effector and non-effector pathways as determined by genomic alterations influence drug sensitivity, and key targeting nodes in specific pathways may yield better therapeutic predictions by pathway-centric rather than gene-centric approaches. We propose a model to provide a more comprehensive understanding of pathway-centric cancer therapies, which requires future in vitro and in vivo studies to validate the genes predicted to be affecting drug sensitivities. Our study demonstrates how pharmacogenomic profiling could be used to shed light on pathway-centric personalized cancer therapies.

## Additional file

Additional File 1:
**Table S1.** Genes whose genomic alterations associated with drug sensitivity (Mann–Whitney U test, adjusted *p* < 0.1).

## Abbreviations

CGP: Cancer Genome Project in Wellcome Trust Sanger Institute
CCLE: The Cancer Cell Line Encyclopedia
